# Describing qualitative research undertaken with randomised controlled trials in grant proposals: a documentary analysis

**DOI:** 10.1186/1471-2288-14-24

**Published:** 2014-02-18

**Authors:** Sarah J Drabble, Alicia O’Cathain, Kate J Thomas, Anne Rudolph, Jenny Hewison

**Affiliations:** 1 Medical Care Research Unit, School of Health and Related Research (ScHARR), University of Sheffield, Regent Street, Sheffield S1 4DA, UK; 2 Leeds Institute of Health Sciences, University of Leeds, 101 Clarendon Road, Leeds LS2 9LJ, UK

**Keywords:** Qualitative research, Randomised controlled trials, Writing grant proposals, Documentary analysis

## Abstract

**Background:**

There is growing recognition of the value of conducting qualitative research with trials in health research. It is timely to reflect on how this qualitative research is presented in grant proposals to identify lessons for researchers and research commissioners. As part of a larger study focusing on how to maximise the value of undertaking qualitative research with trials, we undertook a documentary analysis of proposals of funded studies.

**Methods:**

Using the *meta*Register of Controlled Trials (*m*RCT) database we identified trials funded in the United Kingdom, ongoing between 2001 and 2010, and reporting the use of qualitative research. We requested copies of proposals from lead researchers. We extracted data from the proposals using closed and open questions, analysed using descriptive statistics and content analysis respectively.

**Results:**

2% (89/3812) of trials in the *m*RCT database described the use of qualitative research undertaken with the trial. From these 89 trials, we received copies of 36 full proposals, of which 32 met our inclusion criteria. 25% used less than a single paragraph to describe the qualitative research. The aims of the qualitative research described in these proposals focused mainly on the intervention or trial conduct. Just over half (56%) of the proposals included an explicit rationale for conducting the qualitative research with the trial, the most frequent being to optimise implementation into clinical practice or to interpret trial findings. Key information about methods, expertise and resources was missing in a large minority of proposals, in particular sample size, type of analysis, and non-personnel resources. 28% specifically stated that qualitative researchers would conduct the qualitative research.

**Conclusions:**

Our review of proposals of successfully funded studies identified good practice but also identified limited space given to describing the qualitative research, with an associated lack of attention to the rationale for doing the qualitative research and important methodological details. Acknowledging the space restrictions faced by researchers writing grant proposals, we suggest a starting point for providing practical guidance to help researchers write proposals and research commissioners assess proposals of qualitative research with trials.

## Background

It is becoming more common to undertake qualitative research in conjunction with randomised controlled trials in health research. Researchers have described a wide variety of uses for qualitative research in trials of complex interventions at different stages of the trial e.g. before, during and after a trial [1,2]. For example, it can be used before a trial to develop or refine an intervention so that the optimum intervention is tested in a full trial [3]. It can be used during a full trial in the context of a process evaluation to understand how the intervention was delivered in practice and thereby help to explain why an intervention was effective or not as effective as hoped for [4]. It can be used after a trial to understand how the results of the trial are received by significant stakeholders [2,5]. We have built on this understanding of how qualitative research can be used with trials by developing a framework of how qualitative research has actually been used with trials [6]. Our framework, based on 296 journal articles reporting the qualitative research undertaken with trials, consists of five aspects of a trial that the qualitative research addressed: the intervention content and delivery; the trial design, conduct and processes; the outcomes of the trial; measures of process and outcome used in the trial; and the health condition the intervention was aimed at within the trial. We also identified the potential value of using qualitative research with trials. For example, it could potentially result in more efficient trials by improving recruitment practices [7], increase the validity of trials by ensuring that the right instruments are used to measure important outcomes [8], explain how a trial intervention was effective through exploration of recipients’ views [9], and facilitate the transferability of trial findings to other settings by describing and drawing attention to the impact of the context in which the intervention operated within the trial [10,11].

The use of qualitative research with trials may be related to a growing understanding of the complexity of interventions and the need to use a range of methods within their evaluation. Qualitative research is also used with trials of drugs and devices, which are not classed as complex interventions, because researchers perceive complexities related to the trial conduct, the environment in which the trial takes place, or the patient group that the intervention is aimed at [12]. Researchers may also use qualitative research because they perceive that some funding agencies want a mixed methods approach, or they may use focus groups with experts in the field and potential users of an intervention to develop an intervention and to determine its acceptability in principle or in practice. Indeed funding agencies or research commissioners exert considerable influence on how research is undertaken because they make decisions about which studies to fund based on detailed descriptions within grant proposals. Given the potential value of qualitative research to trials, and its additional cost to that of the trial, it is important that research commissioners have the information they need within grant proposals to select studies for funding that can deliver their potential value.

Each aspect of a complex research proposal needs to include a clear justification for its contribution to answering the main research question, or its potential ‘added value’. This is particularly important for commissioned research which requires researchers to respond to an a priori research brief. In addition, each element of the research proposal must be backed up with a sound description of the research methods proposed. The National Institutes of Health in the United States have presented guidance on writing proposals for mixed methods studies generally rather than specifically to qualitative research undertaken with trials [13]. Major funders in the United Kingdom (UK), such as the National Institute for Health Research (NIHR) and the Medical Research Council, do not currently offer specific guidance on the way qualitative research should be described within an application for trial funding.

A search of the literature also revealed few articles related to either writing proposals for qualitative research in general or specifically related to writing proposals for qualitative research undertaken with randomised controlled trials. Sandelowski and Barroso [14] refer to the process of writing qualitative research proposals as ‘artful design’ which requires ‘reflexivity, elegant expression, imaginative rehearsal, and strategic disarmament’ in order to neutralise any anticipated concerns reviewers’ may have (p.819). They argue that the proposal must demonstrate the researchers’ knowledge of the methods and field whilst demonstrating awareness and respect for their audience. Connelly and Yoder [15] identify a number of common failings in qualitative proposals such as inadequate explanation of methodological techniques, lack of rationale for the use of qualitative methodology, failure to outline the significance of the proposed qualitative study, and underestimation of the costs of undertaking qualitative research. In a study of proposals of mixed methods studies in health research, O’Cathain, Murphy, and Nicholl [16] identified that key aspects of the qualitative research, such as sampling and analysis, were often not described in proposals or described inadequately, highlighting the difficulties commissioners may face when deciding on the quality of the proposed research. As part of a wider study focusing on how to maximise the value of combining qualitative research with randomised controlled trials [12], we undertook a documentation review of grant proposals of trials which included qualitative research to identify how qualitative research is presented for funding in terms of the aims, methods, and the stated relationship of the qualitative research to the trial question in order to contribute to the debate regarding how to maximise the value of undertaking qualitative research alongside or within randomised controlled trials.

## Methods

This research was part of a larger study consisting of four components: a systematic mapping review of journal articles reporting qualitative research undertaken with trials; a documentation review of the proposal and final report of studies combining qualitative research with trials funded in the UK; a survey of the lead investigators of trials funded in the UK to identify qualitative research not visible when searching trials databases; and qualitative interviews with researchers who were involved in studies combining qualitative research with trials. This paper draws on the research proposals from the documentation review.

### Identifying trials with qualitative research

The *meta*Register of Controlled Trials (*m*RCT) http://www.controlled-trials.com/, set up in 1998, is a searchable database containing information about the study hypothesis, design, funders, and contact details of predominantly ongoing trials. We limited our search to trials funded by UK funding bodies because the commissioning of research is likely to be dependent on the country in which it is funded and we wanted to provide guidance for UK commissioners of research and researchers. In 2011, we searched the sub-databases of *m*RCT related to UK-funded trials which were ongoing between 2001 and 2010: UK Trials (UK), Medical Research Council (UK), NIHR Health Technology Assessment (UK), and the Wellcome Trust (UK). The search terms used to identify qualitative research are listed below. Due to the limited search facility in this database, we entered each term individually and then removed any duplicates: qualitative study, qualitative studies, qualitative interview(s), qualitative research, qualitative method(s), qualitative analysis, qualitative analyses, qualitatively analysed, qualitatively analyzed, qualitative data, qualitative approach, qualitative evaluation, qualitative case study, qualitative case studies, qualitative case-study, qualitative case-studies, qualitative inquiry, qualitative exploration, qualitative intervention, qualitative, semi structured interview(s), semistructured interview(s), semi-structured interview(s), in-depth interview(s), interview(s) AND theme(s), interview(s) AND audio recorded, interview(s) AND audio-recorded, descriptive case study, descriptive case studies, focus group(s), focus-group(s), mixed method(s), mixed-method(s), process evaluation, ethnography, ethnographic, ethno methodology, ethnomethodology, phenomenological, action research, content analysis, narrative analysis, grounded theory, thematic analysis, conversation analysis, discursive, discourse analysis, social constructionist, social construction, social constructionism. Where plurals are indicated with (s), this constituted a separate search in the mRCT database.

We identified 122 studies from 3,812 UK trials listed in the different registers. From these we excluded seven trials which were too old (anticipated end date before 2001) and 26 where the search term ‘qualitative’ did not relate to qualitative research, leaving 89 trials with a qualitative component. The lead researcher of each study was contacted via email and a request made for the full proposal, the final report, and a list of any relevant publications in peer-reviewed journals.

### Defining a ‘good’ proposal in this context

There are no agreed quality criteria for assessing the description of qualitative research undertaken with a trial within a grant proposal. We drew on the wider literature about how to write qualitative research proposals [14] and mixed methods proposals [13] and how to assess the quality of mixed methods proposals [16]. We identified the importance of transparency of the research aim, study design, methods, expertise and resources available, as well as communication of why it was important to undertake the study in the first place. For mixed methods studies, we noted the importance of attending to balance between describing the qualitative and quantitative parts of a study in relation to the study aims [13], and integration of the qualitative and quantitative components [13,16].

### Data extraction and analysis

We devised a data extraction form which consisted of closed and open questions. We extracted general information such as the funder, the year the proposal was written, the trial, and the type of intervention. Then we extracted the stated aim and the rationale for undertaking the qualitative research; the qualitative study design, methods and participants; and information about how the qualitative research was described within the structure of the proposal, for example, how much space was given to the description, details of costings and resources, and the description of the relationship between the qualitative research and the trial. The stated aim of the qualitative research, the rationale for including qualitative research, and the language used to describe the relationship between the qualitative research and the trial were extracted verbatim. Data from the closed questions were entered into IBM SPSS Statistics 19 and analysed by the proportion of proposals within each category of each item. The chi-squared test or Fisher’s exact test was used to compare different types of proposals. The open questions were analysed by reading the verbatim extracts and undertaking content analysis. SJD applied the framework from our systematic mapping review [4] to the stated aims of the qualitative research in the proposals. Extra sub-categories were added to this framework because some aims in the proposals were general and could not be categorised, and some aims identified ways of using qualitative research which had not been identified using published articles.

## Results

### Description of studies

We obtained documents for 41/89 studies (46%). These included 36 grant proposals and 17 published protocols. We excluded published protocols because they were written after researchers obtained the funding. On reading the grant proposals, we excluded four because they did not report the use of qualitative research. Of these, three had had qualitative follow-up studies added into the *m*RCT database at a later date, and one was from a study that used the term ‘qualitative’ in the database but there was no evidence of qualitative methods in the proposal. This left 32 proposals for analysis.

The proposals had anticipated start dates between 1999 and 2010 and addressed a wide variety of interventions over a range of topics including conditions such as cancer and mental health, treatments such as complementary medicine, public health issues like obesity and diabetes, and the delivery of health care. The studies were funded by the Health Technology Assessment (n = 19), the Medical Research Council (n = 5), the Department of Health (n = 3), and others including the Scottish Executive Health Department and the Wellcome Trust (n = 5).

### Stated aim and rationale of the qualitative research

All proposals stated at least one aim of the qualitative research and some proposals stated more than one aim; for example different aims for the start-up and main phase of the trial. We identified 67 stated aims in total within the 32 proposals (Table 1). The majority of stated aims focused on the intervention, with 40 of the 67 stated aims relating to the intervention either solely (27 aims) or in combination with other categories (13). Most of these aims were about acceptability and implementation of the intervention either specifically (8) or more generically (7) including feasibility and usefulness of the intervention. The other large intervention sub-category was a generic aim to identify experiences and views of the intervention (11) without further detail about why this might be useful. Two of the aims were unclear about which aspect of the intervention they would focus on. Other stated aims addressed trial design, conduct, and processes (14) in terms of recruitment and retention, experience of participating in a trial, acceptability of the trial in principle or in practice, ethical conduct, and patient and public involvement; outcomes (5) relating to the breadth of outcomes being measured and variation in outcomes between different groups; measures used in the trial (2); and the disease or medical condition (3). Three proposals were unclear about the aim of the qualitative research. Many stated aims were general, for example *to explore patients' views and experiences of [intervention],* but some were specific such as *gain insights into reasons for lack of adherence*.

**Table 1:** **The stated aim of qualitative research described in proposals**^**1,2**^

**Aspect of trial**^**1**^	**Sub-category**^**1**^	**Examples of summarised stated aims of the qualitative research from proposals**
Intervention content and delivery (40)	Intervention development (3)	To develop the [intervention]
		Focus group [] to identify the range of possible interventions
	Intervention components (3)	One of the subsidiary aims is to find out what “support as usual” means
		Identify components of the intervention which contribute to its effectiveness
		Describe “usual care” for this patient group
	Models, mechanisms and underlying theory development (1)	To better understand women’s decisions regarding [intervention]
	Feasibility and acceptability of intervention in practice (8)	Examine acceptability of [intervention] for those over 75 and to ascertain the views of various stakeholders
		Treatment acceptability and usefulness of intervention
		Assess the acceptability of the intervention to patients and healthcare providers
		Explore factors associated with success or failure of the intervention: feasibility, acceptability of different models of [intervention]
	Intervention fidelity, reach & dose (3)	Identify patients’ reasons for completing or not completing [intervention]
		Gain insights into reasons for poor uptake and lack of adherence
		Compliance with intervention
	Intervention implementation (2)	[Healthcare professionals]: experiences of learning and applying new [intervention], their impressions of the ‘climate’ within the group and the impact of the group on the wider service. Managers: Understanding of service policies and practices for [treatment group] – perceived influence that [this type of trial] have had on the clinical practice within each of the services
		Assess the impact of the new [intervention] on the [] workforce, other ‘key’ stakeholders and national leads
	*Generic acceptability / implementation (7)*	*Identify factors (organisational, professional and patient related) that influence successful implementation*
		*Views of [healthcare professionals] concerning implementation of the service. Understand how the intervention worked in practice*
	*Experiences and views about the intervention (11)*	*Main trial – assess experience of receiving [intervention]*
		*To qualitatively explore participants’ experiences of the two [interventions]*
		*Explore patients’ views and experiences of [intervention]*
		*Understand how patients make sense of their treatment and recovery and whether there are any differences in experience between the two treatment groups*
		*Service users: experiences of participating in [intervention]*
		*Patient experiences about the process and effects of [intervention]*
	*Unclear (2)*	*Identify additional factors influencing the uptake of [intervention] and the way it is used*
		*Explore how [intervention] influences beliefs and behaviours*
Trial design, conduct and processes (14)	Recruitment and retention (3)	Development of training programme with individual feedback for staff involved in recruitment
		Start-up: assess parent and clinician attitudes to recruitment methods
		Reasons for rates of recruitment to the trial
	Trial participation (4)	To ascertain the impact of the trial on participants
		Start-up: assess parent and clinician attitudes to participation
		Understand why some [participants] consent to randomisation or express strong preferences for a particular treatment
	Acceptability of trial in principle (1)	Explore attitudes towards a possible [] type of randomised trial; focus groups with patients to explore their attitudes towards the proposed trial
	Acceptability of trial in practice (2)	Understand the reasons for acceptance or refusal of randomization
	Ethical conduct (2)	Ethical and practical issues of consent and assent e.g., merits and problems associated with a number of models of consent, feasibility and acceptability of taking advance consent / assent for research trial procedures
		Consent and assent
	*Public and patient involvement (1)*	*Phase 1: engage service users and carers in driving the research process, and to elicit views of NHS services*
	*Unclear (1)*	*The empirical investigation of the social organisation, production and effects of the RCT in practice*
Outcomes (5)	Breadth of outcomes (2)	Ensure the most relevant [outcome] factors are assessed by the questionnaires
		To access important aspects of [] care not reflected in standardised measures of clinical outcomes
	Variation of outcomes (2)	To examine the perspectives of participant and professional stakeholders using qualitative methods. This is important to understand and explain any differences in outcome between intervention sites
		Phase 2: Determine user's and carers' views on the process and effects of [intervention] compared with the views of those who received the attention control
	*Unclear (1)*	*Start-up: assess parent and clinician attitudes to outcomes*
Measures of process and outcome (2)	Completion of measures (1)	Ensure the feasibility of daily assessment
	Development of measures (1)	Look at ways of asking about [outcome measures]. Ensure that the most relevant [outcome measures] are assessed by the questionnaires
Target condition (3)	Experience of the disease, health behaviour and beliefs (3)	Main trial: explore parent and clinician attitudes and knowledge to [health behaviour]
		Explore issues related to [disease]
*Unclear (3)*	*Unclear (3)*	*Understanding of the processes underlying the changes in patients’ beliefs and attitudes*
		*A qualitative assessment of patient and carer perceptions*
		*Process evaluation: perceived impact of the intervention on outcomes*

^1^Numbers in brackets represent the number of incidences that this category or sub-category was mentioned in the proposals we analysed. All text in square brackets has been removed / summarized to maintain anonymity.

^2^The table is based on a framework developed from a systematic mapping review of articles reporting qualitative research undertaken with trials [6]. Categories in italics are additional categories identified in the proposals.

In terms of the rationale for doing the qualitative research, 18 proposals (56%) included some statement about why it was important to do the qualitative research and its value for the trial (Table 2). Of those 18 proposals, thirteen (72%) gave one rationale whilst five gave more than one rationale. The most common rationales were optimising implementation into clinical practice (6), interpreting the trial findings (5), optimising the trial process (4), improving recruitment and consent procedures for the trial (2), and generating theories and models to guide intervention development (2).

**Table 2:** **The rationale for doing the qualitative research described in proposals**^**1**^

**Rationale**	**Summarised statements from proposals and protocols**
Patient voice or engagement (1)	Engage service users in driving research process. Giving users a ‘voice’ in the evaluation of a health technology of which they will be the recipients
Optimise the trial process / Develop the best processes to maximise the success of the trial (4)	Optimise overall trial process
	Phase 1: Develop qualitative model to understand perceptions and inform strategies for full trial
	Phase 2: Modify trial procedures and documentation in feasibility phase
Improve recruitment and consent procedures for main trial (2)	Development of training programme with individual feedback for staff involved in recruitment. Recommend recruitment strategies most likely to promote recruitment into the main trial
	To pilot and develop trial procedures including modeling consent procedures for main trial
Generate theories and models to guide intervention development (2)	Build conceptual model of [] preferences that will be explored in a subgroup of randomised [participants]
	Gain an insider’s perspective from which a theoretical framework regarding subjective experience of service users can be developed
Generate theories to guide the trial and health community (1)	Develop theoretical model of HTA practice / Develop a critical understanding of social processes and practices implicated in development, implementation and dissemination of a RCT in the field of HTA
Optimise implementation into clinical practice (6)	Inform future development of services of this intervention
	Process evaluation will provide important generalizable information for wider health community about acceptability [in service]
	Inform the roll out of the intervention to the wider community
	Inform commissioners and service providers to contribute to maximisation of quality and uptake of [intervention]
	Assess the feasibility of delivering [intervention] in NHS
Interpret trial findings (especially unexpected findings) (5)	Understand and explain any differences in outcome between intervention sites
	Insight into possible explanations for differential success of intervention
	Interpret trial results to understand why intervention did work / work to further interpret and illuminate the findings from the trial itself
	Influence the interpretation of the outcome data / identify unanticipated outcomes and barriers to change
Other (5)	Understand, as well as quantify, the process and outcome of care
	Bring together the views of different research participants
	Explore range of resource use for economic analysis
	Provide new insights into patients’ views and experiences of [intervention] and usual care
	Provide a richer understanding of patient and carer perceptions to complement quantitative data

^1^Numbers in brackets represent the number of incidences that this category or sub-category was mentioned in the proposals we analysed. All text in square brackets has been removed / summarized to maintain anonymity.

### The presentation of qualitative research in proposals

In this section we consider how the qualitative research appeared in the body of the proposal in terms of how much space was allocated to the qualitative aspects of the study, the methods utilised, and expertise and resources.

#### Space allocated to the qualitative research

Proposals ranged from 4 to 73 pages, with a mean of 23 pages. Based on reading each of the proposals, the amount of space dedicated to the qualitative research in the proposal was categorised as one sentence, more than one sentence but less than a paragraph, more than one paragraph but not in its own section, or having its own section with its own heading. In 8/32 proposals (25%), where less than one paragraph was given, often the same information or sentence was repeated in several sections of the proposal. In four proposals (12.5%) there was more than one paragraph about the qualitative research, whilst in 20 of the proposals (62.5%) there was a section dedicated to the qualitative research, although the quality of what was written varied across proposals. This was not simply that short proposals had short descriptions of the qualitative research. The shortest proposal included a separate section on the qualitative research.

## Methods

Methods were described in 30/32 proposals (94%). Interviews were the most common method, used in all but one study, followed by focus groups, sometimes in combination with interviews. Two studies also used observations and one study also used diaries. 21 studies (66%) included some information about how many interviews and/or focus groups were planned. Of these, ten proposals stated up to 50 interviews, nine studies proposed between 50 and 100, and two studies intended to undertake between 150 and 200 interviews. For those combining interviews and focus groups, two studies proposed up to six focus groups and 15 to 30 interviews, whilst one study proposed 24 focus groups and 60 interviews.

Nine proposals (28%) did not include any mention of analysis. Proposals which did describe the analysis simply stated the type of analysis they would use. The most popular types of analysis were constant comparative analysis (19%), thematic analysis (19%), content analysis (13%), and framework analysis (9%), with interpretative phenomenological analysis mentioned in one proposal. The eight proposals which gave less than one paragraph for the qualitative research were less likely to include an analysis section (Fisher’s exact test, p = 0.002), with half of them including a description of the type of analysis to be employed.

### Expertise and resources

75% of proposals included some information about who would do the research, of which nine proposals (28%) stated that they would use qualitative researchers, with another 11 (34%) stating that they would employ researchers but not specifying whether they were qualitative researchers, or stating that the trial or project managers or the lead researcher would do the qualitative research, and four proposals (13%) not providing information. One proposal stated that they would train community advisers to do the qualitative research.

We only received 11 proposals with any details of costs, and of those, we could identify the qualitative costings for three proposals. In terms of non-staff resources, 19/32 proposals (59%) included information about resources to undertake the qualitative research. These included transcription (53%), travel (47%), equipment (26%), and training (21%).

#### Language describing the relationship of the qualitative research to the trial

20/32 proposals (63%) described a relationship between the qualitative research and the trial. Four of these simply used the terms ‘with’ or ‘also’; for example, ‘we will *also* conduct semi-structured interviews’, or ‘an RCT *with* a qualitative evaluation’. Those studies giving less space in the proposal to describe the qualitative research were less likely to express this relationship (Fisher’s exact test, p = 0.01). Of the remaining 16 studies, 10 described a relationship which placed the qualitative study as subordinate to the trial, represented by language such as ‘embedded’, ‘incorporating’, ‘nested’, ‘sample from’, and ‘sub-study’. The language used to express a subordinate role often implied a degree of integration with the trial with terms such as ‘embedded’, ‘nested’ and ‘incorporated’. The other six proposals used language such as ‘alongside’, ‘concurrent’, ‘in combination with’, ‘linked’, and ‘parallel’. Some of these terms suggested some form of integration such as ‘linked’, ‘in combination with’ and ‘mixed methodology framework’, whereas others signified more separation such as ‘alongside’, ‘parallel’, and ‘concurrent’.

## Discussion

Of 3,812 UK trials on a trials database, we identified 2% (89) using qualitative research. Although 63% of the 32 relevant grant proposals we obtained contained a section dedicated to the qualitative research, a large minority described the qualitative research in less than a paragraph. Key information could be missing including the rationale for undertaking the qualitative research and methodological details such as sample size and approach to analysis. This was similar to issues previously identified by Connelly & Yoder for qualitative research proposals [15] and O’Cathain et al. [16] for mixed methods research proposals such as a lack of rationale for the use of qualitative methodology, and a failure to outline the significance of the proposed qualitative study. Given that these were the proposals of successfully funded studies, this raises concern that research commissioners may be making judgements about funding qualitative research with trials based on limited information.

All proposals stated the aim of the qualitative research, which was often directed at the trial in terms of the intervention and the trial conduct. This reflected the categories identified by O’Cathain and colleagues. However, there were some differences. In our research proposals in the intervention content and delivery category did not contain research about perceived value and benefits of the intervention or acceptability of the intervention in principle. Whilst in the trial design, conduct and processes category, adaptation of trial conduct to local context, and impact of trial on staff, researchers or participants were absent. We did find one proposal relating to public and patient involvement which was not included in O’Cathain et al.’s [6] framework. The most common reasons stated for conducting the qualitative research related to the acceptability and implementation of the intervention. Interestingly, in one proposal the qualitative research was aimed at engaging trial participants in the research process. Many stated aims were general, for example *to explore patients' views and experiences of the intervention,* while some were much more specific, such as *assess the acceptability of the intervention to patients and healthcare providers*, *describe ‘usual care’ for this patient group,* or *gain insights into reasons for lack of adherence to the intervention*. The strength of the more specific aims was that the purpose of the qualitative research was transparent; indicating that the researchers had given thought to the aspect of the trial endeavour that there was uncertainty about. The more general aims might imply a flexible approach, open to emerging findings, or alternatively a lack of thought on the part of researchers about the aspects of the trial it was important to explore. Whilst a general aim may be fine in the hands of an experienced researcher, we suggest that it is insufficient to allow research commissioners to make informed decisions about the value of qualitative research.

Of particular concern was the absence of any rationale for conducting the qualitative research with the trial in 44% of proposals. Sandelowski and Barroso [14] state that for qualitative research proposals, although there is a tension between the emergent nature of the qualitative research and the planning of the research, ‘the significance of establishing significance cannot be overestimated. A research proposal low in significance, albeit high in technical perfection, is not likely to be funded’ (p.783). However, a proposal with the stated aim of finding out about participants’ experiences can allow for important insights to emerge provided it is conducted well, thus still providing value for the trial. What we are trying to caution against is a lack of thought about the importance of the qualitative research in the context of a trial, or its inclusion due to fad or funders’ request without much recourse to the reasons for undertaking it or how it could help the trial.

We recognise that researchers can be constrained by the necessity of providing detailed information about how the trial will be conducted and the lack of space available on application forms. However, the amount of space available in proposals overall did not correspond to the amount of space given over to describe the qualitative research. There were important gaps in how the methods were described. Sample size was too often missing, leaving research commissioners not knowing how many interviews and focus groups they were funding. We also noted that the number of interviews and focus groups proposed in the proposals seemed extremely high when one considers the amount of analysis involved, highlighting a potential feasibility issue for some studies in completing the planned data collection or analysing a large volume of data successfully. This may be a particular problem in large scale trials where samples need to be relatively large to represent all the different groups within the whole trial, although research suggests that other mixed methods studies also struggled with this issue [16]. It may be important for funders to question whether the qualitative data is being subject to quantitative objectives such as generalizability when samples are being proposed. Researchers writing qualitative research for a trial audience may feel that they must conform to the quantitative terminology and methodology of the trial. However, for qualitative proposals to be successful, whilst researchers must think about their audience, decisions about data collection should be based on qualitative interpretations of reliability and validity without being either offensive (dismissing the trial methodology) or defensive (stating limitations inappropriate for qualitative research such as generalisability) [14].

Whilst it may be difficult to know exactly who will do the research, and some projects may use qualitative researchers although it is not stated in the proposal, the use of trial staff to undertake the qualitative research suggests that the qualitative research may be undertaken without the required expertise. In the case of the trial manager undertaking the qualitative research, this may be subsumed by the requirements to deliver the trial. Additionally, in terms of resources, only 31% of proposals with qualitative research mentioned transcription as a resource requirement or gave enough information on the proposal for this to be identified, even when costs were provided. Overall, the lack of information about costs and resources means that it may be difficult for funders to decide whether the qualitative research is adequately resourced, whether it is feasible to complete, or if it provides value for money.

Only half of the proposals described any clear relationship between the qualitative research and the trial. Unsurprisingly, the qualitative research was mostly described as subordinate to the trial. This could influence why the qualitative research methodology received less focus in the grant proposal than the trial. Hesse-Biber [17] argues that this subordinate language reveals implicit positivistic assumptions being applied to the research in which the trial is more valued and important than the qualitative research. However the language used to express a subordinate role in our study often implied a degree of integration with the trial with terms such as ‘embedded’, ‘nested’ and ‘incorporated’. Where the qualitative work was given a more equal footing, there was a mixture of language suggesting some form of integration such as ‘linked’, ‘in combination with’, and language that signified more separation such as ‘alongside’ and ‘parallel’. We suggest that subordination or equality between the qualitative research and the RCT may not be an issue in itself if there is sufficient integration between the qualitative research and the trial. Integration although often difficult to achieve [18] can be beneficial in a number of ways such as improving the cohesiveness of the trial team, improving what we understand about how trials operate, and enabling analysis and interpretation of trial results. We believe that the relationship between the qualitative research and the trial, and in particular the level of integration between the two, should be given some thought within the proposal so that it is understood by the team before the trial commences.

### Limitations of the research

This research was limited to UK proposals and to UK funding bodies and may not be generalizable to other countries, although we believe it is also likely to be of interest to others outside the UK. The response rate to requests for proposals was low. This may have been due to the age of our trials and the out of date contact details for lead researchers on older trials in the database we used. The number of proposals we included was reduced further because we excluded published protocols because they were deemed to be written after the funder had approved the proposal; however we did use documents entitled protocols in our sample, which seemed similar in style and content to the named proposals we received. Our sample of 32 was relatively small and we cannot be sure how representative they were of all proposals. However, from the experience of members of our team who have been involved in approving proposals for these types of studies, the issues identified in this paper were recognised as regularly occurring in trial proposals featuring qualitative research.

A further limitation of this research was that we did not make a direct comparison between the qualitative and quantitative aspects of the proposal. It may be that some issues, such as lack of information about the expertise of researchers, applied to both the trial and the qualitative research within proposals. O’Cathain et al. [16] compared the qualitative and quantitative components of proposals in another study and found that the qualitative component was much less likely to be adequately described. We did consider the amount of space given to the qualitative research in the whole proposal. Many proposals consisted of pages about the trial and a paragraph or less about the qualitative research. An extreme example was a 73 paged proposal with just a couple of paragraphs describing the qualitative research. We are not suggesting that the qualitative research should be given the same amount of space as the trial but we are advocating that if researchers are seeking money for it, then adequate detail is required to allow research commissioners to make informed decisions about funding.

### Guidance for researchers and commissioners

Whilst we found some examples of good practice, we believe there is a need to improve how qualitative research is described in proposals combining qualitative research and trials. The need to improve proposals of mixed methods research more generally has already been identified in the UK [16] and the United States [13]. Researchers wishing to apply best practice for writing proposals combining trials and qualitative research could make use of the excellent guidance developed for mixed methods research for a major funding body in the United States [13], which is relevant to all types of studies and addresses the whole study. We would like to propose some guidance which is aimed at the qualitative research only in the specific scenario of it being undertaken with a trial. Based on our observations in this study, we suggest that this specific guidance would be useful for those writing and reviewing proposals so that researchers can plan and communicate their qualitative research and research commissioners can make more confident decisions about what research to fund and whether it provides value for money. Table 3 presents a starting point for such guidance.

**Table 3:** Guidance for researchers and commissioners on writing proposals for the qualitative research undertaken with trials

Aim	Describe the aim of the qualitative research. Where appropriate identify aims specific to the trial e.g. ‘to explore patient views on adherence to the trial intervention’ rather than using general aims e.g. ‘to explore patient experiences’.
Rationale	Describe the rationale for including qualitative research; identify areas of uncertainty to be explored. Include a statement addressing the ways in which the aims of the qualitative research will ‘add value’ to the trial.
Methods	Provide a clear account of the proposed methods of data collection including the location and timing of data collection, and the skills and seniority of the person who will undertake data collection.
	Describe the sample frame, sampling method(s), and sample size. Where the sample frame is trial participants, specify whether intervention, control or both will be included.
	Describe and reference the proposed approach to analysis. A rationale for the approach to be taken may be included.
	Identify the qualitative research skills and seniority of the person who will undertake the analysis and write-up.
Integration with trial	Outline suggestions for integrating and synthesising qualitative data / findings with the trial results.
Costs	Describe the full costs of the qualitative research and highlight any dedicated equipment, software, staff, and transcription costs.
Leadership	Identify which of the co-applicants will take overall responsibility for the qualitative research and describe their role in the design, data collection, analysis and write-up of the study.

## Conclusions

Our review of proposals of successfully funded studies identified a lack of important details about the qualitative research with the implication that funders are sometimes making decisions based on inadequate information. Acknowledging the space restrictions faced by researchers writing grant proposals, we suggest practical guidance (Table 3) to help researchers to write proposals and research commissioners to assess proposals of qualitative research with trials.

## Competing interests

The authors declare that they have no completing interests.

## Authors’ contributions

AOC and KT designed the study. AOC, KT and JH obtained the funding. SJD wrote the first draft of this paper. AR collected the data. SJD, AOC and KT analysed the data. All authors contributed to the interpretation of the findings and the editing of the article. All authors read and approved the final manuscript.

## Pre-publication history

The pre-publication history for this paper can be accessed here:

http://www.biomedcentral.com/1471-2288/14/24/prepub
